# Supplementary material for: TastepepAI: An artificial intelligence platform for taste peptide de novo design
Source: PLoS Comput Biol. 2025 Oct 16;21(10):e1013602. doi: 10.1371/journal.pcbi.1013602 (PMC12543283; doi:10.1371/journal.pcbi.1013602)
Supplement: S1 File — (ZIP) [file pcbi.1013602.s006.zip › Supplementary Data 2/TaPep73 HPLC.doc]

**HPLC REPORT**

| Sample: | NJP93380 TaPep73 AQ-5 | | | Analyzed date: | 2024-9-25 |
| --- | --- | --- | --- | --- | --- |
| Analyst: | LY Reconstitution: 1mg/0.5ml H_2_O | | | | |
| Lot. No.: | P240920-MX1172309 | | | | |
| Column: | 250*4.6mm,ChromCore 120 C18 5um | | | | |
| Solvent A | A: 0.1% Trifluoroacetic Acid in 100% Acetonitrile | | | | |
| Solvent B | B: 0.1% Trifluoroacetic Acid in 100% Water | | | | |
| Gradient: |  | A | B |  | |
|  | 0.0min | 11% | 89% |  | |
|  | 25.0min | 36% | 64% |  | |
|  | 25.1min | 100% | 0% |  | |
|  | 30.0min | Stop | |  | |
| Volume: | 10μl | | |  | |
| Wavelength: | 220nm | | |  | |
| Flow rate: | 1.0ml/min | | |  | |
| ───────────────────────────────────────────────────  Peak No Ret Time Height Area Conc.  ───────────────────────────────────────────────────  1 6.663 204.528 1127.100 0.0127  2 7.382 438.523 4679.101 0.0527  3 9.533 11378.749 207038.516 2.3329  4 9.883 467219.094 8539795.000 96.2268  5 9.883 4757.021 113608.781 1.2801  6 11.433 305.507 3488.545 0.0393  7 24.522 585.151 4911.300 0.0553  ───────────────────────────────────────────────────  Total 100.00 | | | | | |
